# Intimate partner violence and women's mental health during the COVID-19 pandemic in Brazil

**DOI:** 10.47626/2237-6089-2022-0594

**Published:** 2024-11-26

**Authors:** Angelica Cerveira de Baumont, Géssica Sá Oliveira, Juliana Bastos de Figueiredo, Júlia Foschiera dos Santos, Bruna Pasqualini Genro, Luísa Fernanda Habigzang, Gisele Gus Manfro

**Affiliations:** 1 Universidade Federal do Rio Grande do Sul Hospital de Clínicas de Porto Alegre Porto Alegre RS Brazil Programa de Transtornos de Ansiedade, Hospital de Clínicas de Porto Alegre (HCPA), Universidade Federal do Rio Grande do Sul (UFRGS), Porto Alegre, RS, Brazil.; 2 UFRGS Departamento de Psiquiatria Porto Alegre RS Brazil Programa de Pós-Graduação em Psiquiatria e Ciências do Comportamento, Departamento de Psiquiatria, UFRGS, Porto Alegre, RS, Brazil.; 3 Serviço de Bioética Diretoria de Pesquisa Porto Alegre RS Brazil Serviço de Bioética, Diretoria de Pesquisa (DIPE), HCPA, Porto Alegre, RS, Brazil.; 4 Pontifícia Universidade Católica do Rio Grande do Sul Porto Alegre RS Brazil Pontifícia Universidade Católica do Rio Grande do Sul (PUCRS), Porto Alegre, RS, Brazil.

**Keywords:** Domestic violence, gender-based violence, depression, suicidal ideation, coronavirus

## Abstract

**Objectives:**

Intimate partner violence (IPV) increased extensively around the world during the pandemic, causing severe harm to women's mental health. However, there are no studies showing these effects in Brazil. The objectives of this study were to assess perpetration of IPV and presence of depression and suicidal ideation in women living in Brazil during the coronavirus disease 2019 (COVID-19) pandemic.

**Methods:**

Cross-sectional online survey including women living in Brazil from July 2020 to June 2021. Participants answered a 43-item self-administered questionnaire exploring their characteristics and life changes due to the pandemic (CoRonavIruS Health Impact Survey [CRISIS]), IPV (World Health Organization Violence Against Women [WHO-VAW]), and depressive symptoms or suicidal ideation (Patient Health Questionnaire-9 [PHQ-9]). We used Poisson multiple regression analyses with robust variance to model associations between IPV and mental health outcomes, considering aspects of social vulnerability as covariates.

**Results:**

We found high frequencies of IPV (33.3%), depression (36.1%), and suicidal ideation (19.8%) among the participants. IPV was significantly associated with depression (prevalence ratio [PR] = 1.502, p = 0.001 for one type of IPV; PR = 2.702, p < 0.001 for two or three types of IPV) and with suicidal ideation (PR = 2.264, p < 0.001 for one type of VPI; PR = 3.272, p < 0.001 for two or three types of IPV). Food insecurity, being black, lower educational levels, and being in a relationship with a person of the same gender were associated with one or both mental health outcomes.

**Conclusions:**

We demonstrated associations between IPV and higher frequencies of depression and suicidal ideation in women living in Brazil during the COVID-19 pandemic, highlighting the urgency of strengthening strategies to protect women during adversity.

## Introduction

Intimate partner violence (IPV) is a complex topic that involves issues related to the patriarchal system, responsible for the existence of power asymmetry in interpersonal relationships.^1^ IPV is defined as any behavior by a partner or ex-partner that causes physical, psychological, or sexual harm, which can include both physical aggression and sexual coercion, as well as psychological abuse and controlling behaviors.^2^ It is considered multifactorial, with different determinants involved such as cultural issues and gender inequalities, leading to severe consequences for women's lives.^3^ In addition to the individual harm, IPV also significantly affects the whole of society with economic burden and causes threat and deprivation to offspring.^1^ The World Health Organization (WHO) points out that IPV is the most common type of violence against women in the world, affecting 27% of women in their lifetime, and is unequivocally a public health problem.^4^

Mental health problems, emotional distress, depression, anxiety, posttraumatic stress disorder (PTSD), substance abuse, and even suicidal behavior are common problems among women who are victims of violence.^5-7^ The effects on women's mental health can be acute or chronic, with long-term outcomes.^1^

Notably, some social markers increase vulnerability to IPV, such as race (Black women suffer more IPV), social stratum (women in poverty are most vulnerable), and age (women aged 18 to 29 are the most affected).^8-10^ These markers are also associated with more severe mental health outcomes.^11,12^ Moreover, the negative impact of IPV is exacerbated by an absent social support network and difficulty in accessing protection and care networks that guarantee rights and psychosocial care.^9,13^

With the advent of the coronavirus disease 2019 (COVID-19) pandemic, physical distancing was the method found to protect the population's health, reducing the high rate of transmission of the virus. In this context, an increase in violence was observed precisely due to forced coexistence, economic stress, and fears due to the pandemic,^14-16^ associated with the lack of support networks. A systematic review of the effects of the pandemic on IPV showed that this type of violence against women increased extensively around the world during the outbreak.^17^ In Brazil, calls reporting violence against women increased by 17.9% in March and 37.6% in April 2020, compared to the same period in 2019.^18^ Between March and April 2020, cases of femicide in the country increased by 22.2% in 12 states, compared to the same period the year before.^19^ This increase is very worrying, considering that the femicide rate in the country had already increased by 11.3% between 2017 and 2018 and by 7.3% between 2018 and 2019.^20,21^ Interestingly, the states with the most alarming increases in femicide rates are not necessarily the same states with the highest increases in intentional violent deaths between 2019 and 2020 (e.g., Acre and Maranhão).^18,20^

Factors identified as intensifying or precipitating IPV during the COVID-19 pandemic are mainly related to reduced wages, unemployment, lack of resources, female economic dependence, substance use by partners, and the impairment of both informal and institutional support networks.^22,23^ Women subjected to domestic violence during the lockdown were found to have more severe symptoms of depression, anxiety, and stress.^24^

Despite the evidence pointing to a trend of worsening IPV during the pandemic, as well as the impacts of the IPV on victims’ mental health in developed countries, there are few studies showing these effects during the pandemic in middle-income countries. This study aimed to assess perpetration of IPV and the presence of depression and suicidal ideation among women living in Brazil during the COVID-19 pandemic, searching for associations between IPV and these mental health outcomes.

## Methods

### Study design and participants

This study was a cross-sectional online survey conducted among Brazilian women from July 2020 to Jun 2021. The survey was performed using validated instruments hosted on Google forms and the technical functionality of the electronic questionnaire was tested by the researchers before the link was made available.

Participants were recruited through social media (including sponsored links), constituting a convenience sample. We included cisgender and transgender women residing in Brazil and over 18 years of age.

### Instruments

We included 20 questions from the CoRonavIruS Health Impact Survey (CRISIS) (http://www.crisissurvey.org/^25^), a self-administered questionnaire evaluating participant characteristics and life changes due to the pandemic. This questionnaire has been used previously in studies with the Brazilian population.^26,27^

IPV was investigated in participants who were in a current or recent relationship (during the last month) using the WHO Violence Against Women (WHO-VAW) questionnaire. This is a 13-question instrument exploring physical, sexual, or psychological violence perpetrated by women's intimate partners.^28^ It has been validated for the Brazilian population.^29^

We assessed depressive symptoms with the Patient Health Questionnaire-9 (PHQ-9), as validated for the Brazilian population, using a cutoff score greater than or equal to 10 to define presence of important depressive symptoms.^30^ Response to item 9 of this instrument regarding suicidal ideation was a strong predictor of suicide attempt and suicide death.^31,32^

The complete questionnaires are available in Portuguese (original) and in English as Supplementary Material S1.

### Statistical analysis

Sample characteristics were described as means (standard deviations [SD]) or percentages. We estimated frequencies of IPV, depression, and suicidal ideation separately for the first (July to October 2020) and the second (December 2020 to June 2021) waves of increases in COVID-19 cases in Brazil, and for the whole period of data collection. We assessed differences between participants evaluated cross-sectionally for each of the waves and IPV victims’ characteristics using chi-square tests.

We used Poisson multiple regression analyses with robust variance to model associations between IPV (one type of violence and two or three types of violence) and depression (PHQ ≥ 10) or suicidal ideation (any answer other than "not at all" to item 9 of the PHQ-9). We first calculated univariate Poisson regression, prevalence ratios (PR), and 95% confidence intervals (95%CI) for each variable possibly associated with the outcomes. Afterward, any variable that was significant to p < 0.1 in the univariate model was entered in the multiple Poisson regression analyses. Finally, variables with p > 0.05 were excluded one by one for all steps of the multivariate models. We tested all independent variables for multicollinearity in the multiple linear regression and reported the results as PR and 95%CI.

All analyses were performed using Statistical Software for Social Sciences (SPSS) for Windows, version 21.0 (SPSS Inc., Chicago, IL, USA).

### Ethical considerations

Participation was voluntary, and electronic informed consent was made available to each subject who agreed to participate after reading a detailed and clear description of the main purposes of the study. The main Brazilian help contacts for cases of domestic violence or mental suffering were provided to participants at the beginning and end of the form. Considering the participants’ possible vulnerability, participation was anonymous, the questionnaire was brief, and most questions were not mandatory. All the data collected were treated as secret and confidential, stored on a local electronic device, and all records in a virtual or shared environment were erased.

The project was conducted in accordance with current Brazilian regulations and approved by the Ethics Committee of the Hospital de Clínicas de Porto Alegre (CAAE: 33690420.9.0000.5327).

### Availability of data and materials

The datasets used and/or analyzed during the current study is available from the corresponding author on reasonable request.

## Results

### Participant characteristics

A total of 660 women were included, all cisgender, average age 37.3 years (SD = 11.3). Most participants were white (79.6%), residents of urban areas (65.3%), and from the South region of Brazil (74.4%). The sample of participants surveyed in the first wave of the pandemic differed significantly from the second wave in terms of race (higher proportion of black women in the second wave) and Brazilian states represented (increase in proportions from the North, Northeast, and Midwest in the second wave) (Table 1).

**Table 1:** Demographic characteristics of participants

		1st wave sample	2nd wave sample		Total sample
Variables		(n = 349)	(n = 311)	p-value	(n = 660)
Race (%)					
	Black	49 (14.1)^a^	85 (27.4)^b^	**< 0.001**	134 (20.4)
	White	298 (85.9)^a^	225 (72.6)^b^		523 (79.6)
Age, mean (SD)		38.82 (11.29)	36.73 (11.35)	0.091	37.3 (11.36)
Region (%)					
	North	0 (0.0)^a^	8 (2.6)^b^	**< 0.001**	8 (1.2)
	Northeast	8 (2.3)^a^	38 (12.2)^b^		46 (7.0)
	Midwest	1 (0.3)^a^	16 (5.1)^b^		17 (2.6)
	Southeast	39 (11.2)^a^	58 (18.6)^b^		97 (14.7)
	South	300 (86.2)^a^	191 (61.4)^b^		491 (74.5)
Place of residence (%)					
	Large city	235 (67.5)	195 (62.7)	0.381	430 (65.3)
	City outskirts	27 (7.8)	31 (10.0)		58 (8.8)
	Small city/village/rural area	348 (24.7)	311 (27.3)		171 (25.9)
Education (%)					
	Elementary school	10 (2.9)	7 (2.3)	0.391	17 (2.6)
	High school	92 (26.4)	87 (28.0)		179 (27.2)
	Undergraduate	110 (31.6)	81 (26.0)		191 (29)
	Postgraduate	136 (39.1)	136 (43.7)		272 (41.3)
Marital status (%)					
	Married	195 (56.2)	177 (57.8)	0.197	372 (57.0)
	Dating/engaged	55 (15.9)	60 (19.6)		115 (17.6)
	Single/not applicable	97 (28.0)	69 (22.5)		166 (25.4)
Employment (%)					
	Working in person	84 (24.2)	81 (26.4)	0.130	165 (25.2)
	Teleworking/working from home	137 (39.5)	104 (33.9)		241 (36.9)
	Off work	24 (6.9)	13 (4.2)		37 (5.7)
	Loss of employment/unemployment	102 (29.4)	109 (35.5)		211 (32.3)
Current relationship (%)					
	Same gender	7 (2.6)	12 (5.0)	0.167	19 (3.7)
	Other gender	259 (97.4)	230 (95.0)		489 (96.3)

SD = standard deviation.

Each superscript letter denotes a subset of categories whose column proportions do not differ significantly from each other at the 0.05 level.

Bold type denotes statistically significant difference.

Table 2 describes the characteristics of the IPV victims and the three different types of violence. We included women who suffered at least one type of violence in the last month. In summary, black women, those living on city outskirts, and women who have lost their jobs represent the majority of women subjected to physical and sexual violence.

**Table 2:** Characteristics of women who were victims of intimate partner violence (IPV) during the pandemic in Brazil

Variables		Psychological violence (%)	p-value	Physical violence (%)	p-value	Sexual violence (%)	p-value	Any violence (%)	p-value
Race									
	Black	37.7	0.200	10.8	**0.037**	12.4	**0.002**	38.2	0.240
	White	30.9		4.9		3.9		31.9	
Area of residence									
	Small city/village/rural area	33.3	0.175	8.6	**0.001**	9.2*	**< 0.001**	34.1	0.266
	City outskirts	45		18.4*		17.5*		44.7	
	Large city	30.5		3.6*		2.7*		31.7	
Education									
	Elementary school	33.3	0.689	22.2	0.070	2.7	**0.046**	30	0.506
	High school	32.4		8.8		6.4*		37.7	
	Undergraduate	36.2		5		9.4		34.3	
	Postgraduate	30.1		4.5		11.1*		33.3	
Marital status									
	Married	36.1	**0.003**	7.1	**0.023**	5.9	0.621	36.4	**0.011**
	Dating/engaged	21.7		1.6		4.7		23.8	
Current relationship									
	Same gender	41.2	0.435	11.8	0.236	5.2	0.604	41.2	0.600
	Other gender	31.9		5.2		5.9		32.7	
Employment									
	Loss of employment	45.3*	**0.025**	20*	**0.002**	15.1*	**0.009**	46*	**0.029**
	Off work	44.8		7.1		10.7		46.4	
	Teleworking /working from home	31.7		4.3		4.2		32.4	
	Working in person	25.2*		3.7		3		26.1*	

Bold type denotes statistically significant difference.

* Statistically significant association by analysis of adjusted residuals, to a 5% significance level.

### Frequency of IPV and mental health outcomes

Five hundred and eighteen women answered the WHO-VAW questionnaire. Overall, 33.3% of them reported having experienced some type of IPV in the last month: 32.4% psychological violence, 6.1% physical violence, and 5.6% sexual violence (8.5% reported having experienced physical and/or sexual violence).

**Figure 1:**
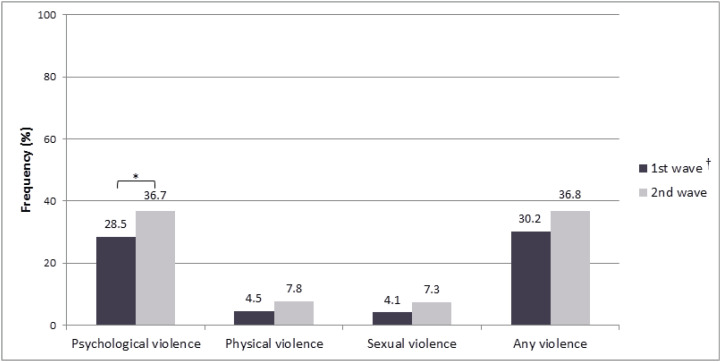
Frequencies of intimate partner violence in women during the COVID-19 pandemic in Brazil (n = 518). * p < 0.05. ^†^ Waves of increases in cases of COVID-19 pandemic in Brazil.

To understand how the increase in the number of cases of COVID-19 and the subsequent increase in physical distancing impacted the occurrence of IPV and mental health in Brazilian women, we examined these variables separately among participants in each pandemic wave of cases. The frequency of psychological violence was higher in the second wave (36.7%) than in the first wave (28.5%, p = 0.047) (Figure 1).

**Figure 2:**
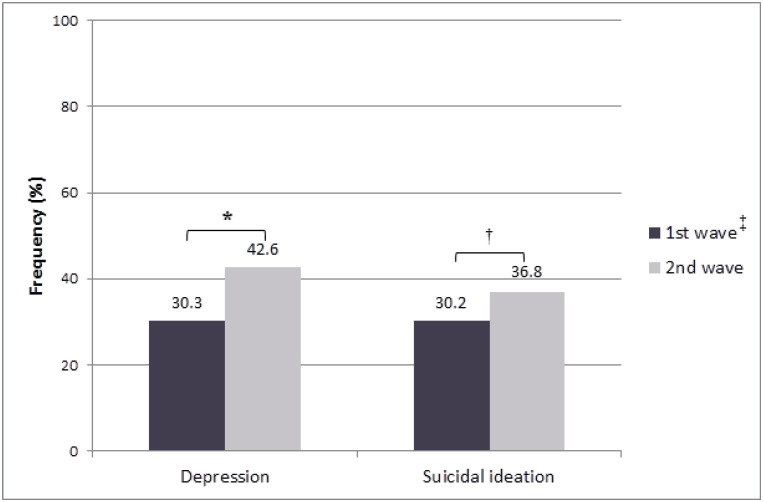
Frequencies of depression and suicidal ideation among women during the COVID-19 pandemic in Brazil (n = 660). * p < 0.01; ^†^ p < 0.001. ^‡^ Waves of increases in cases of COVID-19 pandemic in Brazil.

The frequencies of depression and suicidal ideation among the participants (n = 660) were 36.1% and 19.8%, respectively. Both mental health outcomes were significantly higher among participants who responded during the second wave of the pandemic (Figure 2).

### Association between IPV and mental health outcomes

In the multiple Poisson regression analysis, we can highlight that having suffered one type of IPV was associated with a 50.2% higher frequency of depression, and having experienced two or three types of violence was associated with almost three times the frequency of depression compared to not having suffered any form of IPV. We found four times higher rates of depression in women who had only attended elementary school. Besides, our data showed 55.7% higher rates of depression among women in a same-gender relationship, and 60.4 % higher rates among those in a situation of food insecurity. On the other hand, living with children was associated with a 35.5% lower frequency of depression (Table 3).

**Table 3:** Univariate and multivariate Poisson regression analysis of factors associated with the presence of depression in women during the COVID-19 pandemic in Brazil

Independent variables		Univariate analysis		Multivariate analysis	
		Unadjusted PR (95%CI)	p-value	Adjusted PR (95%CI)	p-value
Number of types of IPV					
	Two or three	2.748 (2.104-3.589)	**< 0.001**	2.702 (1.988-3.673)	**< 0.001**
	One	1.623 (1.236-2.132)	**0.001**	1.502 (1.148-1.966)	**0.003**
	None	1	-	1	-
Marital status					
	Dating/engaged	1.249 (0.966-1.617)	0.090	-	-
	Married	1	-	-	-
Race					
	Black	1.533 (1.241-1.893)	**< 0.001**	-	-
	White	1	-	-	-
Current relationship					
	Same gender	1.947 (1.349-2.810)	**< 0.001**	1.557 (1.053-2.303)	**0.026**
	Other gender	1	.	1	-
Area of residence					
	Small city/village/rural area	1.05 (0.826-1.336)	0.688	-	-
	City outskirts	1.405 (1.044-1.891)	**0.025**	-	-
	Large city	1	-	-	-
Education					
	Elementary school	1.940 (1.066-3.529)	**0.030**	4.208 (2.127-8.325)	**< 0.001**
	High school	2.405 (1.853-3.120)	**< 0.001**	2.445 (1.756-3.403)	**< 0.001**
	Undergraduate	1.657 (1.241-2.212)	**0.001**	1.951 (1.365-2.790)	**< 0.001**
	Postgraduate	1		1	
Housing insecurity					
	Yes	1.636 (1.322-2.024)	**< 0.001**	-	-
	No	1	-	-	-
Food insecurity					
	Yes	1.941 (1.600-2.355)	**< 0.001**	1.604 (1.264-2.036)	**< 0.001**
	No	1	-	1	-
Previous government assistance program					
	Yes	1.462 (1.071-1.997)	**0.017**	-	-
	No	1	-	-	-
Employment					
	Loss of employment	1.802 (1.323-2.453)	**< 0.001**	-	-
	Off work	1.459 (0.962-2.214)	0.075	-	-
	Teleworking/working from home	0.952 (0.706-1.283)	0.745	-	-
	Working in person	1		-	
Living with children					
	Yes	0.700 (0.553-0.885)	**0.003**	0.645 (0.488-0.852)	**0.002**
	No	1	-	1	-

95%CI = 95% confidence interval; COVID-19 = coronavirus disease 2019; IPV = intimate partner violence; PR = prevalence ratio.

Bold type denotes statistically significant difference.

Having suffered one type of IPV was associated with more than twice the frequency of suicidal ideation and having suffered two or three types of violence was associated with more than three times the frequency of this outcome. Black women had a 55.5 % higher frequency of suicidal ideation and those in a situation of food insecurity had a 65.4 % higher frequency of suicidal ideation, whereas living with children reduced the PR for suicidal ideation by 37.4 % (Table 4).

**Table 4:** Univariate and multivariate Poisson regression analysis of factors associated with presence of suicidal ideation in women during the COVID-19 pandemic in Brazil

Independent variables		Univariate analysis		Multivariate analysis	
		Unadjusted PR (95%CI)	p-value	Adjusted PR (95%CI)	p-value
Number of types of IPV					
	Two or three	4.098 (2.686-6.25)	**< 0.001**	3.272 (2.040-5.246)	**< 0.001**
	One	2.369 (1.596-3.517)	**< 0.001**	2.264 (1.538-3.332)	**< 0.001**
	None	1	-	1	-
Marital status					
	Dating/engaged	1.326 (0.904-1.946)	0.148	-	-
	Married	1	-	-	-
Race					
	Black	1.925 (1.409-2.631)	**< 0.001**	1.555 (1.082-2.234)	**0.017**
	White	1	-	1	-
Current relationship					
	Same gender	1.399 (0.645-3.035)	0.396	-	-
	Other gender	1	-	-	-
Area of residence					
	Small city/village/rural area	1.287 (0.914-1.814)	0.149	-	-
	City outskirts	1.460 (0.902-2.362)	0.123	-	-
	Large city	1	-	-	-
Education					
	Elementary school	1.778 (0.716-4.414)	0.215	1.662 (0.721-3.831)	0.233
	High school	2.292 (1.572-3.342)	**< 0.001**	1.905 (1.233-2.943)	**0.004**
	Undergraduate	1.424 (0.933-2.175)	0.102	1.399 (0.87-2.249)	0.166
	Postgraduate	1	-	1	-
Housing insecurity					
	Yes	1.649 (1.199-2.268)	**0.002**	-	-
	No	1	-	-	-
Food insecurity					
	Yes	2.299 (1.703-3.104)	**< 0.001**	1.654 (1.145-2.387)	**0.007**
	No	1	-	1	-
Previous government assistance program					
	Yes	1.385 (0.831-2.309)	0.211	-	-
	No	1	-	-	-
Employment					
	Loss of employment	2.022 (1.290-3.169)	**0.002**	-	-
	Off work	0.892 (0.400-1.986)	0.779	-	-
	Teleworking/working from home	0.848 (0.547-1.315)	0.461	-	-
	Working in person	1	-		
Living with children					
	Yes	0.674 (0.475-0.956)	**0.027**	0.626 (0.431-0.910)	**0.014**
	No	1	-	1	-

95%CI = 95% confidence interval; COVID-19 = coronavirus disease 2019; IPV = intimate partner violence; PR = prevalence ratio.

Bold type denotes statistically significant difference.

## Discussion

In this study, we evaluated the perpetration of IPV against women living in Brazil during the COVID-19 pandemic, searching for associations between IPV and depression or suicidal ideation. IPV was associated with higher frequencies of both mental health outcomes, proportionally to the number of different types of violence suffered.

In agreement with our findings, studies before the pandemic showed a higher prevalence of common mental disorders among women who reported having suffered IPV, according to the severity of violence.^1,33^ Similarly, recent studies assessing the effects of lockdown during the pandemic on domestic violence against women and their mental health reported associations between violence and higher scores for depression, anxiety, stress, and suicidal ideation.^34^ Notably, in Brazilian samples, the female population had stronger associations with depressive symptoms^35,36^ and suicidal ideation^37^ during the COVID-19 pandemic, highlighting the importance of identifying factors that might help explain these findings.

In our study, we observed a higher frequency of depression and suicidal ideation in the sample whose data were collected during the second wave of increases in cases of COVID-19 compared with the sample whose data were collected in the first wave of the rise in cases. The frequency of psychological violence also increased when comparing these groups. It should be noted that these two samples differed in terms of the proportion of black women and also in terms of the distribution across Brazil's regions, which could contribute, at least in part, to the difference reported. Interestingly, findings show a reduction in COVID-19 anxiety over time in Brazil.^38^ Although anxiety and depression are both internalizing disorders, they have different characteristics and manifestations. Therefore, it is important to understand the impact of stressors on each specific disorder and their different symptoms in Brazilian women.

The pandemic exacerbated domestic violence rates worldwide.^14,17^ In agreement with this observation, our study found higher rates (33.3%) of recent IPV among participants compared to the 7.60% rate in Brazil in 2019. According to data from Brazilian state Public Security Secretariats, an alarming increase in cases of femicide was observed when the first quarter of 2020 was compared with the same period in 2019. Contrary to these data, reports of violence against women did not follow this increase, leading to the assumption that coexistence and isolation could affect reporting of offenses against women.^39^ The isolation needed to contain the pandemic, compounded by an absence of effective public policies to combat domestic and family violence, made women more vulnerable and interfered with their access to services and support networks.^14,40^

Victims of psychological violence often do not tell anybody or report the violence because they do not believe the violence they suffered is severe enough. Furthermore, they may also fear threats or aggression against themselves and their families.^41^ In our study, psychological violence was the most frequent form of IPV reported, in agreement with previous studies.^24,42-45^ Similarly, between 2014 and 2015, psychological abuse was the most frequent type of IPV reported in Brazil, accounting for 11.7% of the victims.^46,47^ On the other hand, the physical and sexual violence rates reported here may be underestimated. Many women do not identify experiences such as slaps and shoving as physical violence because they are culturally normalized, and they have a previous history of other physical violence. In addition, beliefs and social gender roles, such as the "marital contract," contribute to many women not perceiving acts of sexual violence as such.^48^

Ten per cent of women worldwide and 3.1% in Brazil have been subjected to physical and/or sexual IPV in the past 12 months.^4,49^ In our study, 8.5% of participants reported experiencing physical and/or sexual violence in the last month. Among these, most were black women living on the city outskirts who had lost their jobs. IPV is associated with social inequalities, with higher frequencies among black and low-income women.^50^ It is worth noting that in Brazil, black race works as a marker of social disadvantage, behaving as a proxy for unfavorable socioeconomic situations.^50^ Similarly, neighborhoods with low income and education levels and high levels of residential mobility and criminality had a higher risk of IPV.^51^ Moreover, economic instability, expressed as unemployment, declining wages, lack of resources, and female economic dependence, precipitated or intensified factors of marital violence during the COVID-19 pandemic.^23^

IPV is a complex phenomenon, with risks linked to interaction of multiple factors on individual, relational, community, and sociocultural levels.^51,52^ For this reason, we chose to perform multivariate analysis including factors that increase women's social vulnerability to better understand the effects of IPV on victims’ mental health. We found higher frequencies of depression and suicidal ideation among women who experienced IPV and those in a situation of food insecurity. Food insecurity has also been associated with IPV and mental health problems like depression, anxiety, panic disorder, and suicidal ideation in previous studies.^53,54^

Likewise, during the COVID-19 lockdown, there were marked reductions in income, increases in food insecurity and IPV, and deterioration in mental health among mothers in Bangladesh.^55^ There is some evidence that mothers may be particularly susceptible to depression while living in poverty and experiencing family stress, including IPV.^56-58^ Notwithstanding, we found an inverse relationship between living with children and the presence of depression or suicidal ideation, suggesting a possible protective factor of motherhood in our sample.

Being black was associated with a higher frequency of suicidal ideation in our study. Black women with a history of severe IPV had an increased risk for mental disorders, including depressive disorders and suicidal ideation,^59^ while white women had 28% lower odds of experiencing IPV.^60^ Furthermore, lower educational level was associated with higher frequency of depression in our multiple regression analyses. Parents with less than a high-school education seems to be a modifiable risk factor for IPV.^60^ Lower educational levels have been associated with mental health impairment during the pandemic,^61^ and high educational levels can reduce the risks of pandemic-related depression, anxiety, and stress in pregnant women.^62^

Besides, being in a relationship with a person of the same gender was also associated with a higher frequency of depression in our sample, in agreement with studies that show a higher risk of mental disorders and suicidal behavior among LGBTQIA+ people.^63,64^ These health disparities may be due to the negative social experiences that LGBTQIA+ people have endured due to their sexual orientation, like suffering internalized homophobia and the perception of social stigma.^65,66^ Moreover, prevailing public policies do not take the characteristics of lesbian and bisexual women into account, hampering their access to protective networks.^67^ In this sense, the "minority stress model" is a valuable model for understanding the internal and external conditions experienced by LGBTQIA+ individuals and the impacts on their mental health.^65^ This theoretical model refers to stressors related to one's minority status, such as being a member of a sexual or racial minority, having a pervasive influence on the mental health of minorities.^68,69^ It proposes risk (such as abuse, violence, homophobia, and discrimination) and protective factors (such as self-acceptance and social support) related to the stress experienced by individuals whose sexual identity is stigmatized.^65^

Although violence is a universal phenomenon, it produces its worst effects in the historically excluded and vulnerable sections of the population, such as black people, women, and LGBTQIA+ groups. The most severe effects of gender-based violence impact black and poor women.^70^ These effects are manifest in areas such as justice, quality health services, psychotherapy, and other network devices.^71^ In this sense, "intersectionality" has been presented as a plausible approach to the study of phenomena such as IPV. This theoretical model focuses on multiple systems of oppression (sexism, racism, classism, heterosexism, etc.), co-producing adversities in the study of health disparities.^72^ This approach requires public policies that address these structural causes of domestic violence,^71^ particularly during adversity, such as in a pandemic context, in which structural gender, race and income inequalities are exacerbated.^73^ In this sense, our study sought to analyze the relationship between IPV and women's mental health from an intersectional perspective, by relating violence to other factors of vulnerability. We were able to demonstrate the importance of social markers such as race, education, sexual orientation, and food insecurity in Brazilian women's vulnerability to gender-based violence.

Our study has some limitations that should be acknowledged. First, we used an online convenience sampling strategy, not based on a random selection, due to the pandemic context. Selection bias should therefore be considered, limiting the generalizability of our results. Also, the characteristics of our sample in terms of race and education might hinder the generalizability of our findings to all women in Brazil. Still, there were significant differences between the samples whose data were collected during the first and second waves of increases in COVID-19 cases. Second, the cross-sectional design does not allow causal inferences. Third, all measures were self-reported by participants, leading to the potential for systematic underreporting or overreporting. However, this format could reduce the likelihood of inaccurate reporting for sensitive issues such as victimization. Finally, we lost many answers about the participants’ ages due to problems with the electronic form, leading to many missing variables that prevented us from using this information in our models.

Nonetheless, to our knowledge, this is the first study to evaluate the associations between IPV against women and depression and suicidal ideation during the pandemic in a middle-income country that faces many socioeconomic constraints, such as high inequality, violence, and unreliable support networks. Our findings could be useful to generate thoughts about IPV and appropriate prevention and intervention strategies, avoiding more drastic consequences for the mental health of victims, especially in a pandemic context.

## Conclusions

In this study, we demonstrated an association between IPV and higher frequencies of depression and suicidal ideation in women living in Brazil during the COVID-19 pandemic. Our findings confirm the urgency of seeking and strengthening strategies to protect women during times of crisis. The high prevalence of IPV represents a public and mental health challenge in Brazil, and points to two needs: (1) implementation in mental health services of psychotherapy protocols with proven effectiveness for women with a history of IPV; (2) investment in the prevention of violence in intimate relationships, through gender equity programs. It is essential that adequate public policies are developed and intensified to combat violence against women, as well as to reduce social inequalities and vulnerabilities.
