# Supplementary material for: Intimate partner violence and women's mental health during the COVID-19 pandemic in Brazil
Source: Trends Psychiatry Psychother. 2024 Nov 26;46:e20220594. doi: 10.47626/2237-6089-2022-0594 (PMC11790106; doi:10.47626/2237-6089-2022-0594)
Supplement: Supplementary file 1 [file 2238-0019-trends-46-e20220594-suppl01.pdf]

Intimate Partner Violence and Women's Mental Health during the COVID-19 Pandemic in Brazil

Questionnaire in English

(Questions 1 to 21 were adapted from the CRISIS - The CoRonavIruS Health Impact Survey)

1. **Please specify your sex:**
    a. Female
    b. Male

2. **Please specify your gender:**
    a. Male
    b. Female
    c. Other ____________

3. **Age: ______________**

4. **What is your color/race?**
    a. White
    b. Black
    c. Yellow
    d. Brown
    e. Indigenous

5. **What is your nationality?**
    a. Brazilian
    b. Other

6. **In which Brazilian state do you live?**
    a. Acre (AC)
    b. Alagoas (AL)
    c. Amapá (AP)
    d. Amazonas (AM)
    e. Bahia (BA)
    f. Ceará (CE)
    g. Distrito Federal (DF)
    h. Espírito Santo (ES)
    i. Goiás (GO)
    j. Maranhão (MA)
    k. Mato Grosso (MT)
    l. Mato Grosso do Sul (MS)
    m. Minas Gerais (MG)
    n. Pará (PA)
    o. Paraíba (PB)
    p. Paraná (PR)
    q. Pernambuco (PE)
    r. Piauí (PI)
    s. Rio de Janeiro (RJ)
    t. Rio Grande do Norte (RN)
    u. Rio Grande do Sul (RS)
    v. Rondônia (RO)
    w. Roraima (RR)
    x. Santa Catarina (SC)
    y. São Paulo (SP)
    z. Sergipe (SE)
    aa. Tocantins (TO)

7. **Which best describes the area in which you live?**
    a. Large city
    b. Suburbs of a large city
    c. Small city
    d. Town or village
    e. Rural área

8. **What is the highest level of education YOU completed?**
    a. Some grade school
    b. Some high school
    c. High school diploma or GED
    d. Some college or 2-year degree
    e. 4-year college graduate
    f. Graduate or professional degree

9. **Has a health or educational professional ever told you that you had any of the following health conditions (check all that apply)?**
    a. Emotional or mental health problems such as Depression or Anxiety
    b. Problems with alcohol or drugs
    c. Intellectual disability
    d. Autism Spectrum Disorder
    e. Learning Disorder

10. **Please specify your relationship to the people in your home (check all that apply):**
    a. Partner/Spouse
    b. Parent(s)
    c. Grandparent(s)
    d. Siblings
    e. Children
    f. Other relatives
    g. Unrelated person

11. **Please specify your partner's gender:**
    a. Male
    b. Female
    c. Other _______________
    d. Not applicable

12. **Please specify your relationship with your partner/partner:**
    a. Marriage
    b. Common-law marriage
    c. Living together
    d. Dating/engagement
    e. Occasional/sporadic relationship
    f. Not applicable

13. **In the 3 months prior to the Coronavirus/COVID-19 crisis in your area, were you working?**
    a. Yes
    b. No

14. **In the 3 months prior to the Coronavirus/COVID-19 crisis in your area, did you or your family receive money from government assistance programs like welfare, Aid to Families with Dependent Children, General Assistance, or Temporary Assistance for Needy Families?**
    a. Yes
    b. No

15. How would you rate your overall Mental/Emotional health before the Coronavirus/COVID-19 crisis in your area?

   a. Excellent
   b. Very Good
   c. Good
   d. Fair
   e. Poor

In the <u>LAST MONTH</u>:

16. … if you had a job prior to the Coronavirus/COVID-19, are you still working? Y/N/Not Applicable

   a. If yes,
      - Are you still going to your workplace? Y/N
      - Are you teleworking or working from home? Y/N
   b. If no,
      - Were you laid off from your job? Y/N
      - Did you lose your job? Y/N
   c. Not applicable

17. … if you partner had a job prior to the Coronavirus/COVID-19, is he still working? Y/N/Not Applicable

   a. If yes,
      - Are you still going to your workplace? Y/N
      - Are you teleworking or working from home? Y/N
   b. If no,
      - Were you laid off from your job? Y/N
      - Did you lose your job? Y/N

18. … to what degree are you concerned about the stability of your living situation?

   a. Not at all
   b. Slightly
   c. Moderately
   d. Very
   e. Extremely

19. … did you worry whether your food would run out because of a lack of money?

   a. Yes
   b. No

20. … has the quality of your relationships with your partner changed?

   a. A lot worse
   b. A little worse
   c. About the same
   d. A little better
   e. A lot better

21. … how stressful have these changes in contact with your partner been for you?

   a. Not at all
   b. Slightly
   c. Moderately
   d. Very
   e. Extremely

(Questions 22 to 34: WHO-VAW)

The next questions refer to your relationship with your partner.

DURING THE PAST MONTH:

22. **Has he insulted you or made you feel bad about yourself?**
    a. Yes
    b. No

23. **Has he belittled or humiliated you in front of other people?**
    a. Yes
    b. No

24. **Has he done things to scare or intimidate you on purpose?**
    a. Yes
    b. No

25. **Has he threatened to hurt you or someone you care about?**
    a. Yes
    b. No

26. **Has he slapped you or thrown something at you that could hurt you?**
    a. Yes
    b. No

27. **Has he pushed or shoved you?**
    a. Yes
    b. No

28. **Has he hit you with his fist or with something else that could hurt you?**
    a. Yes
    b. No

29. **Has he kicked you, dragged you or beaten you up?**
    a. Yes
    b. No

30. **Has he choked or burnt you on purpose?**
    a. Yes
    b. No

31. **Has he threatened to use or actually used a gun, knife or other weapon against you?**
    a. Yes
    b. No

32. **Has he physically forced you to have sexual intercourse when you didn't want to?**
    a. Yes
    b. No

33. **Did you ever have sexual intercourse when you didn't want because you were afraid of what he might do?**
    a. Yes
    b. No

34. **Has he forced you to do something sexual that you found degrading or humiliating?**
    a. Yes
    b. No

(Questions 35 to 44: PHQ-9)

The next questions ask how you have been feeling recently.

Over the last month, how often have you been bothered by any of the following problems?

35. **Little interest or pleasure in doing things**
    a. Not at all
    b. Several days
    c. More than half the days
    d. Nearly every day

36. **Feeling down, depressed, or hopeless**
    a. Not at all
    b. Several days
    c. More than half the days
    d. Nearly every day

37. **Trouble falling or staying asleep, or sleeping too much**
    a. Not at all
    b. Several days
    c. More than half the days
    d. Nearly every day

38. **Feeling tired or having little energy**
    a. Not at all
    b. Several days
    c. More than half the days
    d. Nearly every day

39. **Poor appetite or overeating**
    a. Not at all
    b. Several days
    c. More than half the days
    d. Nearly every day

40. **Feeling bad about yourself — or that you are a failure or have let yourself or your family down**
    a. Not at all
    b. Several days
    c. More than half the days
    d. Nearly every day

41. **Trouble concentrating on things, such as reading the newspaper or watching television**
    a. Not at all
    b. Several days
    c. More than half the days
    d. Nearly every day

42. **Moving or speaking so slowly that other people could have noticed? Or the opposite — being so fidgety or restless that you have been moving around a lot more than usual**
    a. Not at all
    b. Several days
    c. More than half the days
    d. Nearly every day

43. Thoughts that you would be better off dead or of hurting yourself in some way
   a. Not at all
   b. Several days
   c. More than half the days
   d. Nearly every day

44. If you checked off any problems, how difficult have these problems made it for you to do your work, take care of things at home, or get along with other people?
   a. Not difficult at all
   b. Somewhat difficult
   c. Very difficult
   d. Extremely difficult

Questionnaire in Portuguese (Original)

(As questões 1 a 21 foram baseadas no instrumento CRISIS).

1. Por favor, especifique seu sexo:
   a. Feminino
   b. Masculino

2. Por favor, especifique seu gênero:
   a. Feminino
   b. Masculino
   c. Outro _______________

3. **Idade: ________________**

4. Qual sua cor/raça?
   a. Branca
   b. Preta
   c. Amarela
   d. Parda
   e. Indígena

5. Qual sua nacionalidade?
   a. Brasileira
   b. Outra

6. Em que estado você vive?
   a. Acre (AC)
   b. Alagoas (AL)
   c. Amapá (AP)
   d. Amazonas (AM)
   e. Bahia (BA)
   f. Ceará (CE)
   g. Distrito Federal (DF)
   h. Espírito Santo (ES)
   i. Goiás (GO)
   j. Maranhão (MA)
   k. Mato Grosso (MT)
   l. Mato Grosso do Sul (MS)
   m. Minas Gerais (MG)
   n. Pará (PA)
   o. Paraíba (PB)
   p. Paraná (PR)
   q. Pernambuco (PE)
   r. Piauí (PI)
   s. Rio de Janeiro (RJ)
   t. Rio Grande do Norte (RN)
   u. Rio Grande do Sul (RS)
   v. Rondônia (RO)
   w. Roraima (RR)
   x. Santa Catarina (SC)
   y. São Paulo (SP)
   z. Sergipe (SE)
   aa. Tocantins (TO)

7. **Qual dessas opções melhor descreve o lugar que você mora?**
   a. Cidade grande
   b. Periferia de cidade grande
   c. Cidade pequena
   d. Pequeno município/Vilarejo
   e. Área Rural

8. **Qual o maior grau de ensino que você completou?**
   a. Ensino Fundamental
   b. Ensino Médio Incompleto
   c. Ensino Médio Completo / EJA
   d. Ensino Superior Incompleto
   e. Ensino Superior Completo
   f. Pós-Graduação

9. **Algum profissional de saúde ou de educação já disse que você tinha alguma das condições de saúde abaixo (marque todas que já lhe disseram)?**
   a. Problemas emocionais ou de saúde mental como depressão ou ansiedade
   b. Problemas com álcool ou drogas
   c. Deficiência intelectual
   d. Transtorno de espectro autista
   e. Problemas de aprendizado

10. **Por favor, especifique o seu relacionamento com as pessoas que moram na sua casa (marque todas que se aplicam):**
    a. Parceiro(a)/ Companheiro(a)/ Esposo(a)
    b. Pais
    c. Avós
    d. Irmãos/Irmãs
    e. Outros parentes
    f. Pessoas que não são parentes

11. **Por favor, especifique o gênero do seu(sua) parceiro(a)/ companheiro(a)/ esposo(a):**
    a. Masculino
    b. Feminino
    c. Outro _______________
    d. Não se aplica

12. **Por favor, especifique sua relação com seu(sua) parceiro(a)/ companheiro(a):**
    a. Casamento
    b. União estável
    c. Morando juntos
    d. Namoro/noivado
    e. Relação eventual/esporádica
    f. Não se aplica

13. **Nos 3 MESES ANTERIORES à crise do Coronavírus/COVID-19, você estava trabalhando?**
    a. Sim
    b. Não

14. **Nos 3 MESES ANTERIORES à crise do Coronavírus/COVID-19, você ou sua família receberam dinheiro de programas de assistência governamental (Bolsa Família, BPC, LOAS, por exemplo)?**
    a. Sim
    b. Não

15. Como você classificaria sua saúde mental/emocional antes da crise de Coronavírus/COVID-19 na sua região?
   a. Excelente
   b. Muito boa
   c. Boa
   d. Razoável
   e. Ruim

Durante o <u>ÚLTIMO MÊS</u>:

16. ... se você tinha um emprego antes do Coronavírus/COVID-19, você segue trabalhando?
   a. Sim
      - Você segue indo presencialmente em seu local de trabalho?
         1. Sim
         2. Não
      - Você tem trabalhado de casa / à distância?
         1. Sim
         2. Não

   b. Não
      - Você foi afastado do seu emprego?
         1. Sim
         2. Não
      - Você perdeu o seu emprego?
         1. Sim
         2. Não

   c. Não se aplica

17. ... se seu(sua) parceiro(a)/companheiro(a) tinha um emprego antes do Coronavírus/COVID-19, ele(a) segue trabalhando?
   a. Sim
      - Ele segue indo presencialmente em seu local de trabalho?
         1. Sim
         2. Não
      - Ele tem trabalhado de casa / à distância?
         3. Sim
         4. Não

   b. Não
      - Ele foi afastado do seu emprego?
         1. Sim
         2. Não
      - Ele perdeu o seu emprego?
         1. Sim
         2. Não

   c. Não se aplica

18. ... o quanto você está preocupado com a estabilidade da sua situação de moradia?
   a. Nada
   b. Um pouco
   c. Moderadamente
   d. Bastante
   e. Extremamente

19. **... você se preocupou com ficar sem comida em função da falta de dinheiro?**
    a. Sim
    b. Não

20. **... a qualidade do seu relacionamento com seu(sua) parceiro(a)/companheiro(a) mudou?**
    a. Piorou muito
    b. Piorou um pouco
    c. Segue aproximadamente a mesma
    d. Melhorou um pouco
    e. Melhorou muito

21. **...o quão estressantes têm sido para você essas mudanças no contato com seu(sua) parceiro(a)/companheiro(a)?**
    a. Nada
    b. Um pouco
    c. Moderadamente
    d. Bastante
    e. Extremamente

(Questões 22 a 34: WHO-VAW)
As próximas perguntas se referem ao seu relacionamento com seu(sua) parceiro(a)/ companheiro(a).

<u>DURANTE O ÚLTIMO MÊS</u>, seu(sua) parceiro(a)/ companheiro(a):

22. **Insultou-a ou fez com que você se sentisse mal a respeito de si mesma?**
    a. Sim
    b. Não

23. **Depreciou ou humilhou você diante de outras pessoas?**
    a. Sim
    b. Não

24. **Fez coisas para assustá-la ou intimidá-la de propósito?**
    a. Sim
    b. Não

25. **Ameaçou machucá-la ou alguém de quem você gosta?**
    a. Sim
    b. Não

26. **Deu-lhe um tapa ou jogou algo em você que poderia machucá-la?**
    a. Sim
    b. Não

27. **Empurrou-a ou deu-lhe um tranco ou chacoalhão?**
    a. Sim
    b. Não

28. **Machucou-a com um soco ou com algum objeto?**
    a. Sim
    b. Não

29. **Deu-lhe um chute, arrastou ou surrou você?**
    a. Sim
    b. Não

30. **Estrangulou ou queimou você de propósito?**
    a. Sim
    b. Não

31. **Ameaçou usar ou realmente usou arma de fogo, faca ou outro tipo de arma contra você?**
    a. Sim
    b. Não

32. **Forçou-a fisicamente a manter relações sexuais quando você não queria?**
    a. Sim
    b. Não

33. **Você teve relação sexual porque estava com medo do que ele pudesse fazer?**
    a. Sim
    b. Não

34. **Forçou-a a uma prática sexual degradante ou humilhante?**
    a. Sim
    b. Não

(Questões 35 a 44: PHQ-9)
As próximas perguntas se referem a como você tem se sentido recentemente.

Durante o ÚLTIMO MÊS, com que freqüência você foi incomodado/a por qualquer um dos problemas abaixo?

35. **Pouco interesse ou pouco prazer em fazer as coisas**
a. Nenhuma vez
b. Vários dias
c. Mais da metade dos dias
d. Quase todos os dias

36. **Se sentir "para baixo", deprimido/a ou sem perspectiva**
a. Nenhuma vez
b. Vários dias
c. Mais da metade dos dias
d. Quase todos os dias

37. **Dificuldade para pegar no sono ou permanecer dormindo, ou dormir mais do que de costume**
a. Nenhuma vez
b. Vários dias
c. Mais da metade dos dias
d. Quase todos os dias

38. **Se sentir cansado/a ou com pouca energia**
a. Nenhuma vez
b. Vários dias
c. Mais da metade dos dias
d. Quase todos os dias

39. **Falta de apetite ou comendo demais**
a. Nenhuma vez
b. Vários dias
c. Mais da metade dos dias
d. Quase todos os dias

40. Se sentir mal consigo mesmo/a — ou achar que você é um fracasso ou que decepcionou sua família ou você mesmo/a

a. Nenhuma vez
b. Vários dias
c. Mais da metade dos dias
d. Quase todos os dias

41. Dificuldade para se concentrar nas coisas, como ler o jornal ou ver televisão

a. Nenhuma vez
b. Vários dias
c. Mais da metade dos dias
d. Quase todos os dias

42. Lentidão para se movimentar ou falar, a ponto das outras pessoas perceberem? Ou o oposto – estar tão agitado/a ou irrequieto/a que você fica andando de um lado para o outro muito mais do que de costume

a. Nenhuma vez
b. Vários dias
c. Mais da metade dos dias
d. Quase todos os dias

43. Pensar em se ferir de alguma maneira ou que seria melhor estar morto/a

a. Nenhuma vez
b. Vários dias
c. Mais da metade dos dias
d. Quase todos os dias

44. Se você assinalou qualquer um dos problemas das questões 34 a 43, indique o grau de dificuldade que os mesmos lhe causaram para realizar seu trabalho, tomar conta das coisas em casa ou para se relacionar com as pessoas?

a. Nenhuma dificuldade
b. Alguma dificuldade
c. Muita dificuldade
d. Extrema dificuldade
